# Identification of immune-related key genes in the peripheral blood of ischaemic stroke patients using a weighted gene coexpression network analysis and machine learning

**DOI:** 10.1186/s12967-022-03562-w

**Published:** 2022-08-12

**Authors:** Peng-Fei Zheng, Lu-Zhu Chen, Peng Liu, Hong Wei Pan, Wen-Juan Fan, Zheng-Yu Liu

**Affiliations:** 1 Cardiology Department, Hunan Provincial People’s Hospital, No.61 West Jiefang Road, Furong District, Changsha, 410000 Hunan China; 2 Clinical Research Center for Heart Failure in Hunan Province, No.61 West Jiefang Road, Furong District, Changsha, 410000 Hunan China; 3 Institute of Cardiovascular Epidemiology, Hunan Provincial People’s Hospital, No.61 West Jiefang Road, Furong District, Changsha, 410000 Hunan China; 4 Department of Cardiology, The Central Hospital of ShaoYang, No.36 QianYuan Lane, Daxiang District, Shaoyang, 422000 Hunan China

**Keywords:** Weighted gene coexpression network analysis, Ischaemic stroke, Immune cell subtype distribution pattern, Significant modules, Hub genes

## Abstract

**Background:**

The immune system plays a vital role in the pathological process of ischaemic stroke. However, the exact immune-related mechanism remains unclear. The current research aimed to identify immune-related key genes associated with ischaemic stroke.

**Methods:**

CIBERSORT was utilized to reveal the immune cell infiltration pattern in ischaemic stroke patients. Meanwhile, a weighted gene coexpression network analysis (WGCNA) was utilized to identify meaningful modules significantly correlated with ischaemic stroke. The characteristic genes correlated with ischaemic stroke were identified by the following two machine learning methods: the support vector machine-recursive feature elimination (SVM-RFE) algorithm and least absolute shrinkage and selection operator (LASSO) logistic regression.

**Results:**

The CIBERSORT results suggested that there was a decreased infiltration of naive CD4 T cells, CD8 T cells, resting mast cells and eosinophils and an increased infiltration of neutrophils, M0 macrophages and activated memory CD4 T cells in ischaemic stroke patients. Then, three significant modules (pink, brown and cyan) were identified to be significantly associated with ischaemic stroke. The gene enrichment analysis indicated that 519 genes in the above three modules were mainly involved in several inflammatory or immune-related signalling pathways and biological processes. Eight hub genes (*ADM*, *ANXA3*, *CARD6*, *CPQ*, *SLC22A4*, *UBE2S*, *VIM* and *ZFP36*) were revealed to be significantly correlated with ischaemic stroke by the LASSO logistic regression and SVM-RFE algorithm. The external validation combined with a RT‒qPCR analysis revealed that the expression levels of *ADM*, *ANXA3*, *SLC22A4* and *VIM* were significantly increased in ischaemic stroke patients and that these key genes were positively associated with neutrophils and M0 macrophages and negatively correlated with CD8 T cells. The mean AUC value of *ADM*, *ANXA3*, *SLC22A4* and *VIM* was 0.80, 0.87, 0.91 and 0.88 in the training set, 0.85, 0.77, 0.86 and 0.72 in the testing set and 0.87, 0.83, 0.88 and 0.91 in the validation samples, respectively.

**Conclusions:**

These results suggest that the *ADM*, *ANXA3*, *SLC22A4* and *VIM* genes are reliable serum markers for the diagnosis of ischaemic stroke and that immune cell infiltration plays a crucial role in the occurrence and development of ischaemic stroke.

**Supplementary Information:**

The online version contains supplementary material available at 10.1186/s12967-022-03562-w.

## Background

Stroke is a serious disease with high morbidity and mortality. Stroke is a leading cause of lifelong disability in adults worldwide, and ischaemic stroke accounts for more than 80%. With the increasing severity of social ageing, the acceleration of urbanization, the persistence of cardiovascular risk factors and the prevalence of unhealthy lifestyles, the burden of ischaemic stroke is rapidly increasing [1]. Currently, reliable diagnostic methods for ischaemic stroke mainly rely on imaging methods, such as computed tomography (CT) [2] and magnetic resonance imaging (MRI) [3], which are time-consuming and laborious. Meanwhile, the traditional and effective treatment strategy is to carry out drug thrombolysis and interventional thrombolysis or thrombectomy as soon as possible after the occurrence of ischaemic stroke. These treatments not only require immediate treatment and intervention but also are significantly correlated with an increased risk of fatal bleeding, such as intracerebral haemorrhage and gastric bleeding [4]. Therefore, the early diagnosis, prevention and treatment of ischaemic stroke are facing serious challenges. There is an urgent need to further explore potential reliable serum biomarkers significantly correlated with ischaemic stroke.

At present, a large number of studies have suggested that traditional risk factors including hypertension, hyperlipidaemia and hyperglycemia are significantly associated with several diseases, such as cancer [5–7] and ischemic cardiovascular and cerebrovascular diseases [8, 9]. However, in addition to these common cardiovascular risk factors, the role of inflammation or immune related mechanisms in ischemic cardiovascular and cerebrovascular diseases has received more and more attention. Krishnan et al. found that inflammatory cell infiltration can effectively stimulate and lead to a strong immune response, resulting in dysfunction in the immune microenvironment in the central nervous system and ultimately further leading to the deterioration of patients with cerebral ischaemia [10]. Smith et al. also suggested that proinflammatory cytokines, especially interleukin-1 (IL-1), play a key role in the early inflammatory response after ischaemic stroke, and these inflammatory responses are associated with poorer clinical outcomes in patients with ischaemic stroke [11]. In recent years, immunotherapy has become a novel method to treat cancer [12] and cardiovascular disease [13]. In addition, some studies have revealed that immune regulation can effectively delay the progression of ischaemic stroke, restore neurological function and improve the prognosis of patients, further emphasizing the importance of maintaining immune microenvironment homeostasis for protecting the central nervous system [14, 15]. It has been demonstrated that specific inhibitors of IL-1β can delay the progression of atherosclerosis by inhibiting specific inflammatory pathways associated with atherosclerotic plaque formation [16] and effectively reduce the risk of major cardiovascular adverse events and cardiovascular death [17]. Meanwhile, IL-1 receptor antagonists have been found to be effective in reducing peripheral inflammation in acute ischaemic stroke, thereby improving clinical outcomes in these patients [11]. Therefore, in addition to the current conventional treatment methods, immunoregulatory therapy is expected to be a practical alternative treatment method that is worthy of further in-depth research. In recent years, CIBERSORT, a widely used analysis tool, can use RNA-seq data or microarray data to investigate the infiltration pattern of immune cells and evaluate the proportion of 22 types of immune cells in samples [18]. However, few studies investigated the infiltration pattern of immune cells and the identification of immune-related genes in the peripheral blood of ischaemic stroke patients. Therefore, evaluating the infiltration pattern of immune cells in the peripheral blood of ischaemic stroke patients could help further clarify the immune-related molecular mechanism involved in ischaemic stroke.

With the continuous promotion of gene chip technology, weighted gene coexpression network analysis (WGCNA), a powerful systematic biological method used to analyse network relationships and molecular mechanisms, is widely used to analyse massive amounts of gene expression profile data [19]. WGCNA is often used to identify coexpressed gene modules and further explore the relationship between gene modules and interesting sample features [20]. More recently, machine learning has significantly improved the predictive and accuracy value of key genes identified based on microarrays and next-generation sequencing data [21]. The least absolute shrinkage and selection operator (LASSO) regression and support vector machine-recursive feature elimination (SVM-RFE) algorithm are the most widely used machine learning methods to identify key genes [22]. However, few studies have combined WGCNA, LASSO and SVM-RFE to identify the key genes related to ischaemic stroke.

In the current research, the GSE22255 and GSE58294 datasets were used as the training set, and the GSE16561 dataset was used as the testing set; all datasets were downloaded from the Gene Expression Omnibus (GEO). By removing the interbatch differences between the GSE22255 and GSE58294 datasets, the 25% genes with the highest expression variance were selected for a WGCNA. The potential biological functions of the genes in several key modules that were significantly associated with ischaemic stroke were analysed by Gene Ontology (GO) and Kyoto Encyclopedia of Genes and Genomes (KEGG) analyses. The key genes significantly associated with ischaemic stroke were identified by a LASSO regression combined with SVM-RFE methods. Then, we explored the infiltration pattern of immune cells in peripheral blood from ischaemic stroke patients and further calculated the relationship between several key genes and 22 types of immune cells. Meanwhile, the expression of key genes and their diagnostic efficiency were further validated in the training set, testing set and validation samples.

## Materials and methods

### Ischaemic stroke microarray datasets

**Fig. 1:**
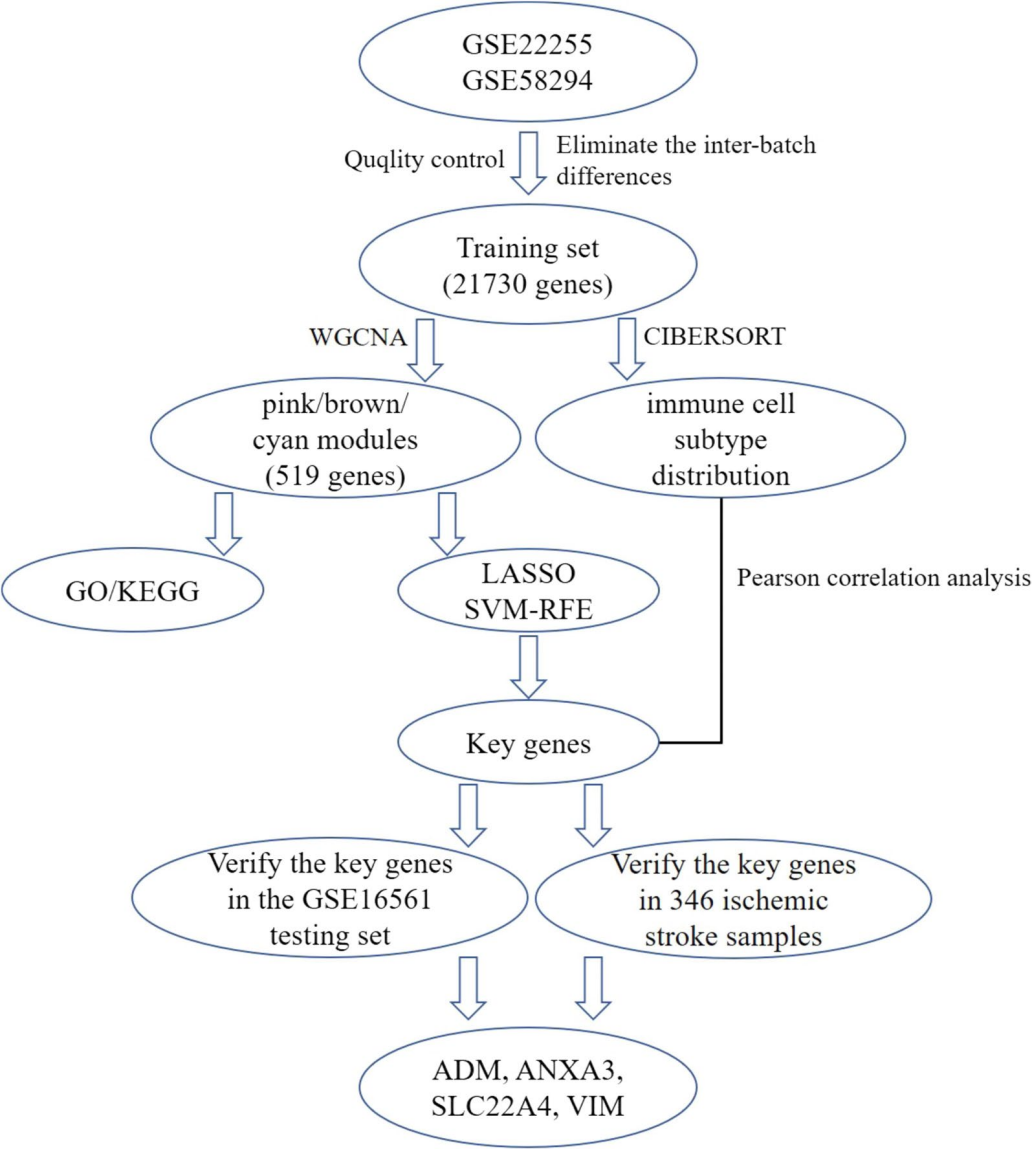
Flow chart of the analysis. GO, Gene Ontology annotation; KEGG, Kyoto Encyclopedia of Genes and Genomes pathway enrichment analyses; LASSO, Least absolute shrinkage and selection operator; SVM-RFE, Support vector machine-recursive feature elimination; WGCNA, Weighted gene coexpression network analysis; *ADM*, Adrenomedullin; *ANXA3*, Annexin A3; *SLC22A4*, Solute carrier family 22 member 4; *VIM*, Vimentin

The gene expression profiles in the GSE22255 (including 20 ischaemic stroke and 20 healthy samples) and GSE58294 (including 69 ischaemic stroke and 23 healthy samples) datasets were extracted from the public database Gene Expression Omnibus (GEO, http://www.ncbi.nlm.nih.gov/geo). The integrated gene expression profile was defined as a training set after normalization and the removal of inter batch differences between GSE22255 and GSE58294. The gene expression profile of GSE16561 was also downloaded from the GEO database as a testing set. The gene expression profiles were normalized using the *normalize Between Arrays* function in the *limma* package [23]. Probes that detected more than one gene were excluded from this study. The expression of genes detected by multiple probes was determined as the average gene expression detected in all probes. Interbatch differences between the GSE22255 and GSE58294 datasets, including 89 ischaemic stroke and 43 healthy samples, were eliminated by the ComBat function in the "sva" R package. The specific workflow is shown in Fig. 1.

### Construction of the WGCNA and identification of modules significantly associated with ischaemic stroke

A critical tool in the study of systems biology is WGCNA, which can construct a gene expression data profile-based scale-free network [24]. The WGCNA method was used to analyse the top 25% of genes with high expression variances. The reliability of the constructed scale-free network is ensured by removing outlier samples. First, a standard-scale free network was used to approximate the appropriate soft threshold power (soft power = 14) before the power function was used to calculate the adjacency values among genes with a variance more significant than all variance quartiles. Then, the adjacency values were transformed into a topological overlap matrix (TOM), and the corresponding dissimilarity (1-TOM) values were derived. Finally, the dynamic tree cut method was used to identify modules by hierarchically clustering genes with 1-TOM as the distance measure, a deep split value of 2 and a minimum size cut-off of 100 for the resulting dendrogram. The relationships between the modules and clinical shapes were evaluated using a Pearson correlation analysis to identify modules of biological significance.

### Enrichment analysis of interesting modules

KEGG and GO enrichment analyses of the genes in the biologically significant modules were carried out by clusterProfler and the DOSE package in R [25]. The threshold was determined to be an FDR < 0.05.

### Construction of the LASSO model and SVM-RFE feature selection process

LASSO and SVM-RFE algorithms were used to identify the key genes with the best prognostic value for ischaemic stroke. A LASSO logistic regression analysis [26] was performed using the "glmnet" package, with the response type set as binomial and alpha set as 1. In addition, SVM-RFE acts as an effective feature selection technique that finds the best variables by deleting the feature vector generated by SVM [27], and the thresholds were set as follows: halve.above = 100 and *k* = 5. Based on the SVM function in the e1071 package of R, the selected biomarkers in the diagnosis of ischaemic stroke were classified and analysed by the SVM classifier. The common genes identified by both machine learning methods were defined as key genes for the subsequent research.

### Evaluation of immune cell subtype distribution

The CIBERSORT.R script downloaded from the CIBERSORT website was utilized to explore the immune infiltration pattern in ischaemic stroke [18]. After obtaining the expression matrix of immune cells according to the instructions of the CIBERSORT website, the “ggplot2” software package was used to draw histograms, heatmaps, and boxplot diagrams. The histogram shows the proportion of 22 infiltrating immune cells in ischaemic stroke patients, and the heatmap and boxplot diagrams show the difference in immune cell infiltration between the control and ischaemic stroke subjects. The "corrplot" software package was used to calculate the Pearson correlation coefficient between each immune cell and display the results in a relevant heatmap.

### Correlation between key genes and immune cells and validation of key genes

The "corrplot" software package was used to calculate the Pearson correlation coefficient between hub genes and each immune cell and display the results in a relevant bar graph. The expression trends of the key genes identified by machine learning in the validation set were evaluated, and the diagnostic accuracy of the key genes was also tested in the training and testing sets.

### Study population

In total, 346 participants, including 166 healthy subjects and 180 ischaemic stroke patients, were recruited from Hunan Provincial People's Hospital. All ischaemic stroke patients underwent detailed and rigorous neurological examinations and brain magnetic resonance imaging (MRI) scans. The diagnostic criteria for ischaemic stroke were based on the International Classification of Diseases (9th Revision). Patients with a history of haematologic, type 1 diabetes, autoimmune, thyroid, neoplastic, renal or liver diseases were excluded. The study protocols were developed based on the Ethics Committee of Hunan Provincial People's Hospital (No: LL-20210615-144) and the 2008 revision of the Declaration of Helsinki of 1975 (http://www.wma.net/en/30publications/10policies/b3/). All subjects provided written and informed consent.

### Diagnostic criteria

The participants were divided into different subgroups based on their alcohol consumption (0 (non-drinker) and ≥ 1 g/day) and smoking status (0 (non-smoker) and ≥ 1 cigarette/day). Hypertension was defined as systolic blood pressure ≥ 140 mmHg and/or diastolic blood pressure ≥ 90 mmHg. Fasting blood glucose ≥ 7 mmol/L was defined as diabetes mellitus. Hyperlipidaemia was defined as TC > 5.17 and/or TG > 1.7 mmol/L.

### RT-qPCR

Fasting venous blood samples of 5 mL were collected from each subject. The total RNA was isolated from isolated peripheral blood monocytes (PBMCs) using TRIzol reagent according to the manufacturer’s instructions. Then, cDNA was reverse-transcribed with a PrimeScript RT reagent kit (Takara Bio, Japan). A Taq PCR Master Mix Kit (Takara) was used to perform quantitative RT‒qPCR based on an ABI Prism 7500 sequence-detection system (Applied Biosystems, USA). The proprietary qPCR primers used in the experiment were designed and validated by Songon Biotech (Songon Biotech, Shanghai, China). A *p *value < 0.05 was considered statistically significant.

### Statistical analyses

SPSS (version 22.0) software was utilized to analyse all data collected in the current research. An independent-samples t-test was used to evaluate whether the continuous data (mean ± SD) were normally distributed between the control subjects and ischaemic stroke patients. The TG levels that were not normally distributed are expressed using the median and quartile ranges and were evaluated using a Wilcoxon−Mann‒Whitney test. Data, such as the sex ratio, the number of smokers, hyperlipidaemia, drinking status, hypertension, and T2DM, were analysed by a chi-square test. The k-fold cross-validation [28] based on logistic regression was used to evaluated the value of areas under the curves (AUCs) in the training set (GSE22255 and GSE58294), testing set (GSE16561) and 346 validation samples. In the process of analysis, ischaemic stroke was defined as the outcome, and each dataset was partitioned randomly into five subsets (k = 5), a single subset was retained as the testing set, and the remaining 4 (k−1) subsets were used as the training set, and repeated 400 times. Then, the average value of AUCs of 2000 replicates (5 × 400) was taken as the calibration value of the AUC of the key genes including *ADM*, *ANXA3*, *CARD6*, *CPQ*, *SLC22A4*, *UBE2S*, *VIM* and *ZFP36*. The bioinformatics analysis and k-fold cross-validation were performed using R software (version 4.1.0). All tests were two-sided, and *p* < 0.05 was considered statistically significant.

## Results

### Data preprocessing

First, the normalized gene expression profiles of the GSE22255, GSE58294 and GSE16561 datasets were obtained after standardizing the data format, adding missing values and removing outliers. Then, after data merging and eliminating the interbatch differences between the GSE22255 and GSE58294 datasets, the combined expression matrix, including 21730 gene symbols, was obtained from the 89 ischaemic stroke and 43 healthy samples in the training set. After removing 4 outlier samples (Additional file 1: Fig. S1), the top 25% of genes with high expression variance in the remaining 128 samples were selected for the subsequent WGCNA and are presented in Additional file 2: Table S1. The gene expression profile of GSE16561 was used as a validation set and is presented in Additional file 3: Table S2. In addition, the disease grouping information of 128 samples is presented in Additional file 4: Table S3.

### Weighted gene coexpression networks

**Fig. 2:**
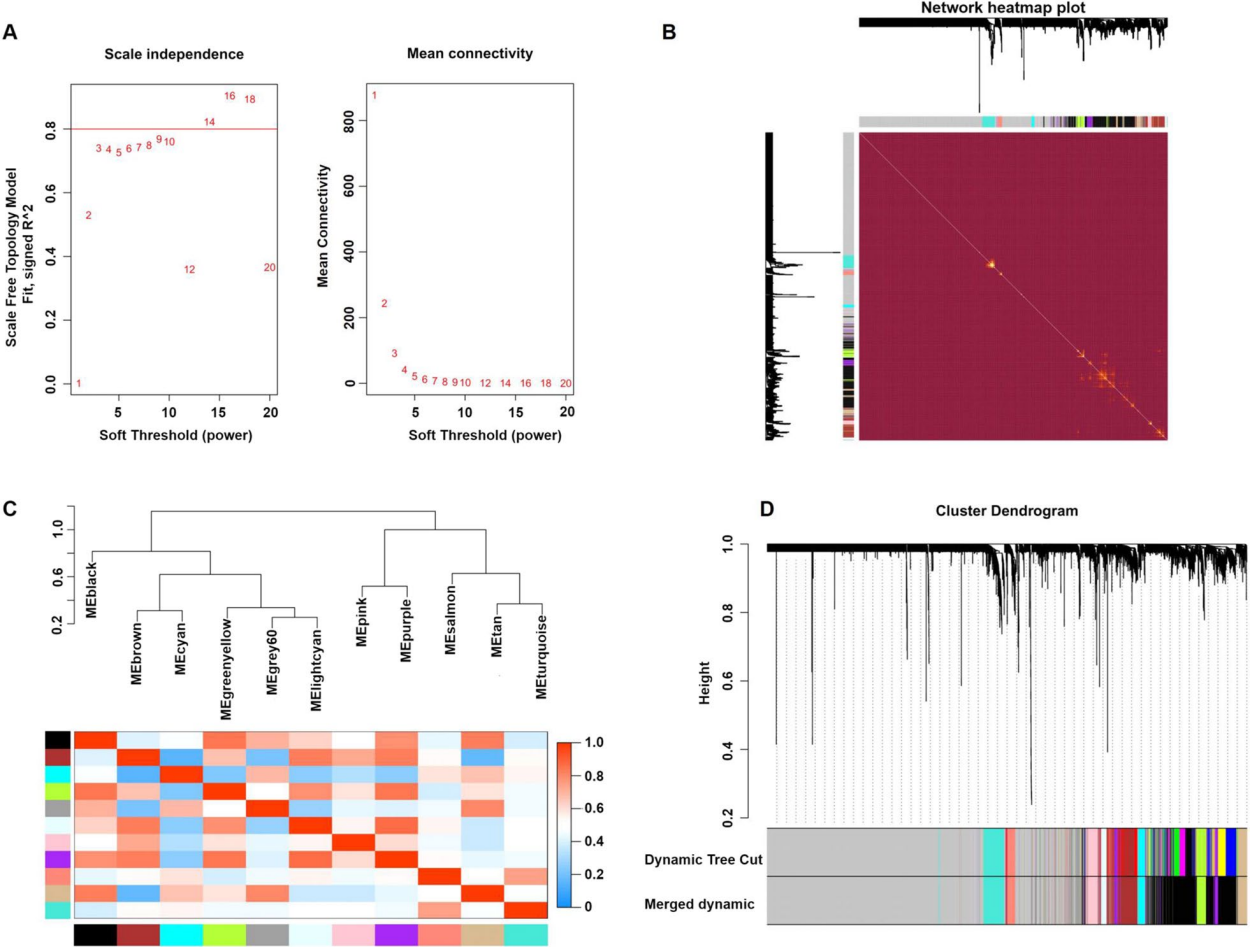
Weighted gene coexpression network analysis. **A** Analysis of the network topology for various soft-thresholding powers. **B** Heatmap of the topological overlap in the gene network. **C** Relationship among all modules. **D** Clustering dendrogram of genes. Gene clustering tree (dendrogram) obtained by the hierarchical clustering of adjacency-based dissimilarity

After the calculation, we revealed that a correlation coefficient greater than 0.8 (the soft threshold β is 14) was highly correlated and suitable for constructing several gene modules (Fig. 2A). A topological overlap matrix (TOM) was constructed by calculating the gene expression profiles' correlation and adjacency matrices. The gene cluster tree is depicted in Fig. 2B. Then, we sought to identify the gene modules of each gene network using the hierarchical average linkage clustering method combined with TOM. Figure 2C depicts the heatmap. The dynamic tree cut algorithm revealed twelve gene modules (Fig. 2D).

### Identification of the modules of interest

**Fig. 3:**
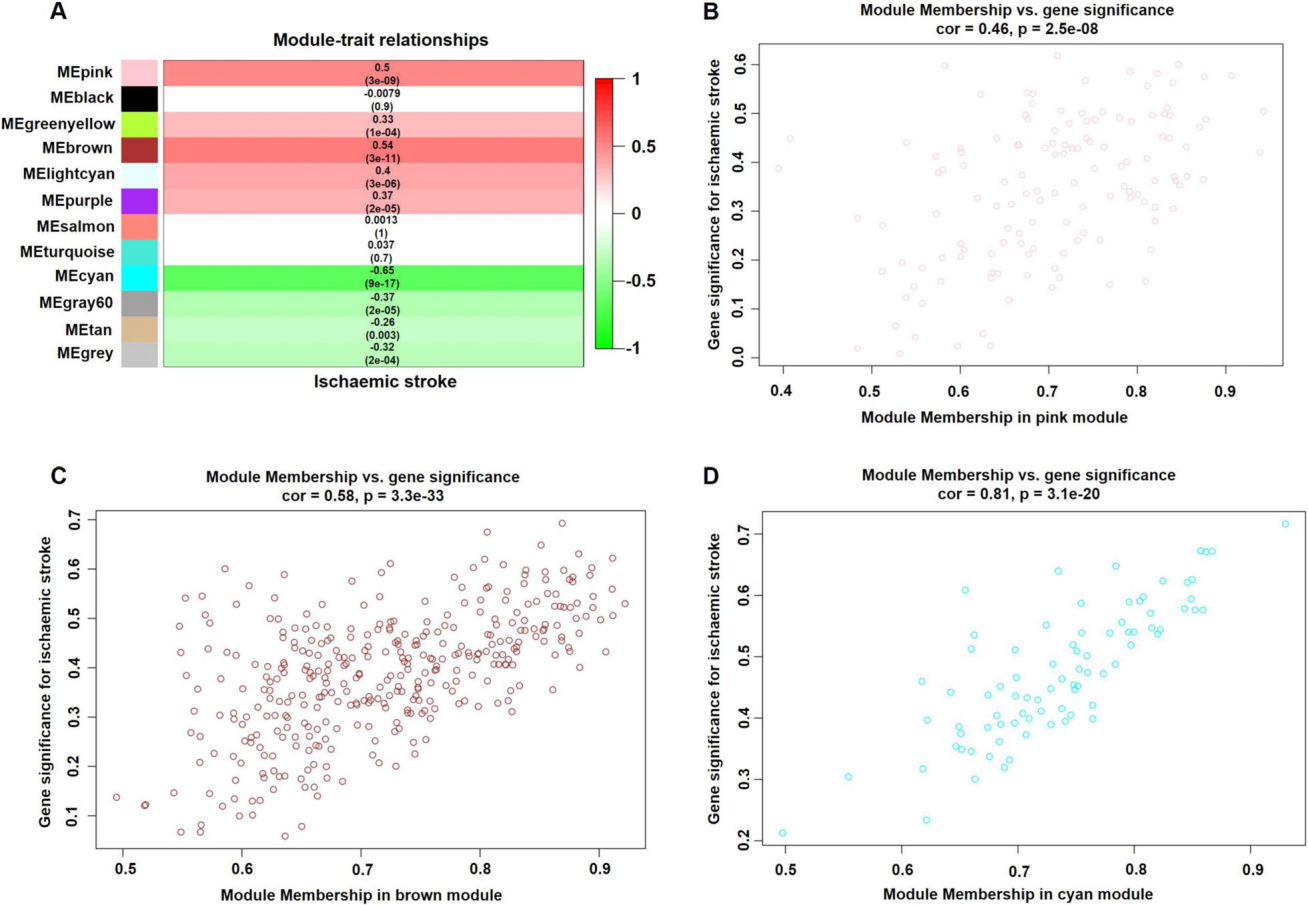
Module-feature associations and associations between gene significance and module membership. **A** Each row corresponds to a modulEigengene, and the column corresponds to the clinical phenotype. Each cell contains the corresponding correlation in the first line and the p value in the second line. The table is colour-coded by correlation according to the colour legend. Scatterplot shows a highly significant correlation between gene significance (GS) versus module membership (MM) with ischaemic stroke in the pink (**B**), brown (**C**) and cyan (**D**) modules

Modules closely related to clinical features are often found to carry important and specific biological significance. As shown in Fig. 3A, the pink (*r *^*2*^ = 0.50, *p* = 3E−09), brown (*r*^2^ = 0.54, *p* = 3E−11) and cyan (*r*^2^ = − 0.65, *p* = 9E−17) modules appeared to be highly correlated with ischaemic stroke. An in-depth calculation was performed to determine the association between the colour of the module and gene significance (GS). The association between the pink module and gene significance was 0.46 (*p* = 2.5E−08) (Fig. 3B), the association between the brown module and gene significance was 0.58 (*p* = 3.3E−33) (Fig. 3C), and the association between the cyan module and gene significance was 0.81 (*p* = 3.1E−20) (Fig. 3D). All gene symbols in the pink, brown and cyan modules, their GS values and corresponding *p* values are described in detail in Additional file 5: Table S4.

### Enrichment analysis of the genes in the pink, brown and cyan modules

**Fig. 4:**
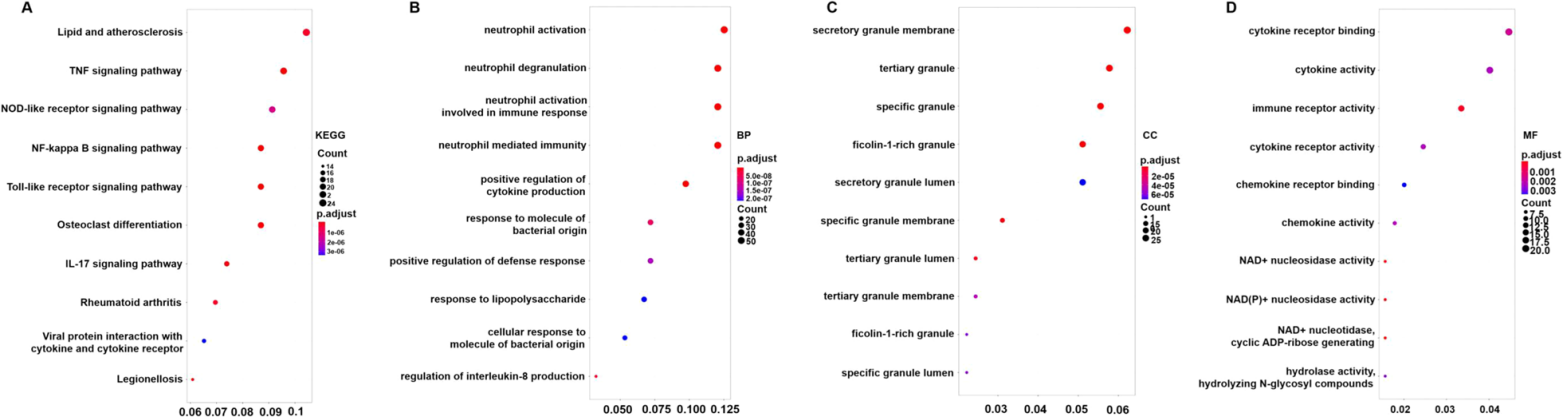
KEGG pathway and GO functional enrichment analyses of genes in the pinkB term is coloured according to the legend. **A** KEGG pathway. **B** Biological process. **C** Cytological components. **D** Molecular function

KEGG pathway and GO enrichment analyses of 519 genes in the pink, brown and cyan modules were carried out to dissect their physiological purposes. Figure 4A shows the top 10 KEGG signalling pathways as follows: hsa05323, rheumatoid arthritis; hsa04064, NF-kappa B signalling pathway; hsa04668, TNF signalling pathway; hsa04620, Toll-like receptor signalling pathway; hsa04621, NOD-like receptor signalling pathway; hsa05134, Legionellosis; hsa04380, osteoclast differentiation; hsa04657, IL-17 signalling pathway; hsa05417, lipid and atherosclerosis; and hsa04061, viral protein interaction with cytokine and cytokine receptor. Figure 4B shows the top 10 biological process as follows: GO:0,002,446, neutrophil-mediated immunity; GO:0,042,119, neutrophil activation; GO:0,001,819, positive regulation of cytokine production; GO:0,043,312, neutrophil degranulation; GO:0,002,283, neutrophil activation involved in immune response; GO:0,002,237, response to molecule of bacterial origin; GO:0,031,349, positive regulation of defence response; GO:0,032,496, response to lipopolysaccharide; GO:0,071,219, cellular response to molecule of bacterial origin; and GO:0,032,677; regulation of interleukin-8 production. These signalling pathways and biological processes are mainly related to inflammation and the immune response. In addition, the cytological components and molecular functions are shown in Fig. 4C, D. The details of these analyses is also provided in Additional file 6: Table S5.

### Identification of hub genes

**Fig. 5:**
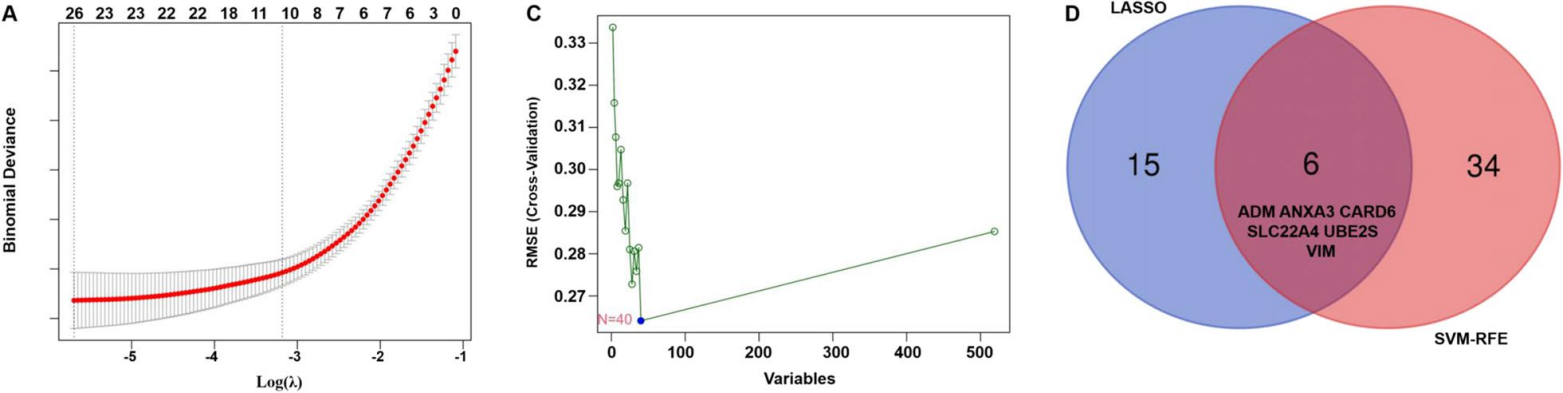
Identification of key genes in ischaemic stroke by machine learning. **A** In total, 25 key genes in ischaemic stroke were identified by a LASSO regression. **B** In total, 40 key genes in ischaemic stroke were identified by the SVM-RFE algorithm. **C** Venn diagram of the genes extracted from the LASSO and SVM-RFE methods. LASSO, least absolute shrinkage and selection operator; SVM, support vector machine-recursive feature elimination

To identify reliable serum biomarkers significantly associated with ischaemic stroke, a LASSO regression and the SVM-RFE algorithm were used to evaluate the characteristic genes in ischaemic stroke based on the gene expression profile of genes in the key modules. The LASSO regression results showed that 21 genes were identified as characteristic genes (Fig. 5A). Meanwhile, in total, 40 genes were identified as key genes by the SVM-RFE algorithm (Fig. 5B). Then, in total, 8 overlapping genes (*ADM*, *ANXA3*, *CARD6*, *CPQ*, *SLC22A4*, *UBE2S*, *VIM* and *ZFP36*) were selected as the core genes for subsequent research (Fig. 5C). In addition, the other genes identified by the LASSO regression and SVM-RFE algorithm are shown in Additional file 7: Table S6.

### Profile of the immune cell subtype distribution pattern

**Fig. 6:**
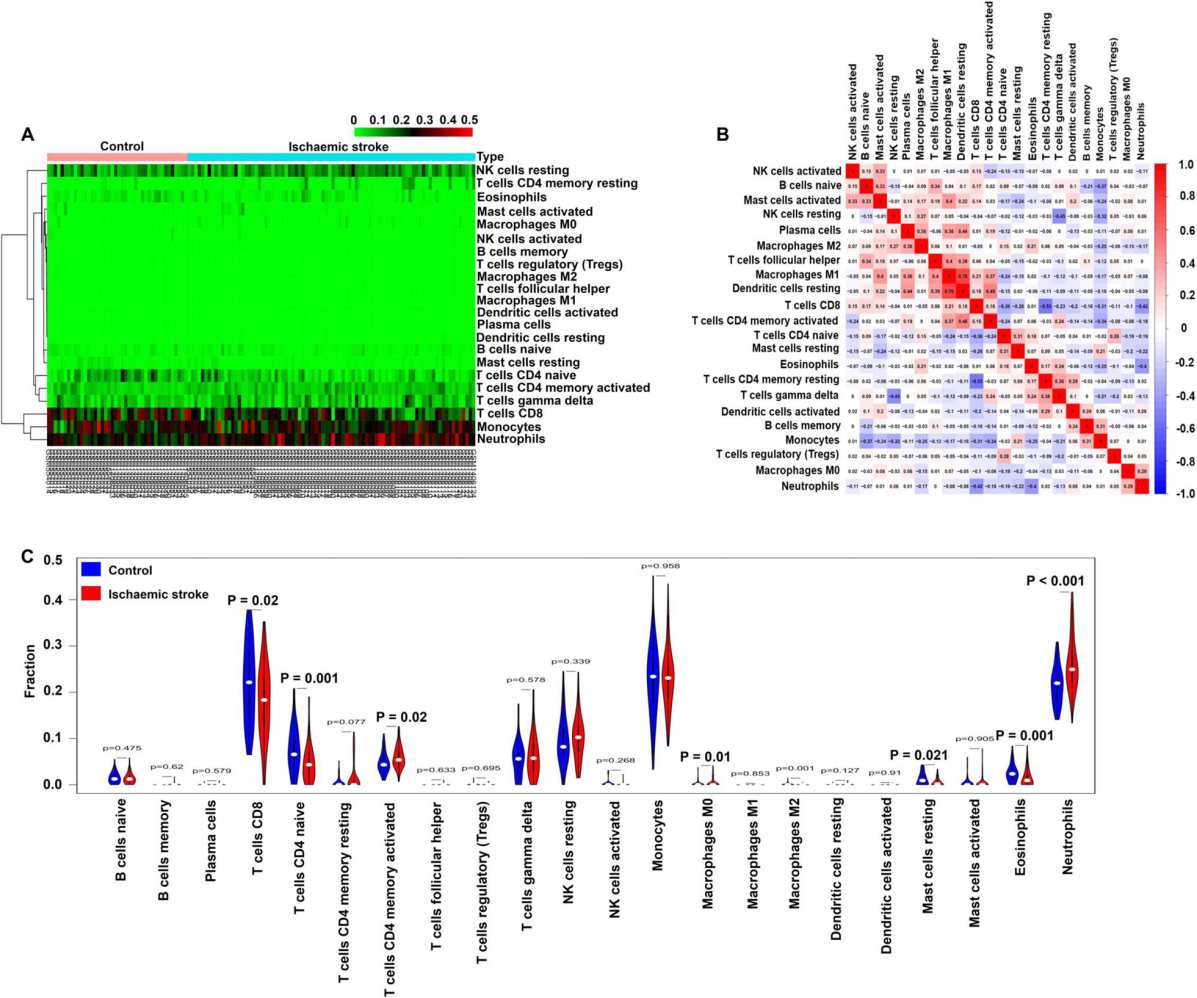
Infiltration pattern of immune cell subtypes in the training set. **A** Heatmap of the 22 immune cell proportions in each sample. **B** Correlation heatmap of all 22 immune cells. **C** Violin plot of all 22 immune cell differentially infiltrated fractions

The CIBERSORT algorithm was utilized to evaluate the differential expression of immune fractions between the control and ischaemic stroke samples. The cumulative histogram visually shows the relative proportion of various immune cell subtypes (Additional file 1: Fig. S2). As shown in Fig. 6A, the heatmap showed that there were significant differences in the proportion of immune cells between the control and ischaemic stroke samples. Using a correlation matrix, we found that neutrophils were negatively correlated with CD8 T cells and eosinophils cells and positively correlated with M0 macrophages (Fig. 6B). Compared with the normal subjects, the ischaemic stroke samples generally contained a decreased infiltration of naive CD4 T cells, CD8 T cells, resting mast cells and eosinophils and an increased infiltration of activated memory CD4 T cells, neutrophils and M0 macrophages (Fig. 6C) (*p* < 0.05–0.01, respectively). In addition, the immune cell infiltration pattern in ischaemic stroke is shown in Additional file 8: Table S7.

**Fig. 7:**
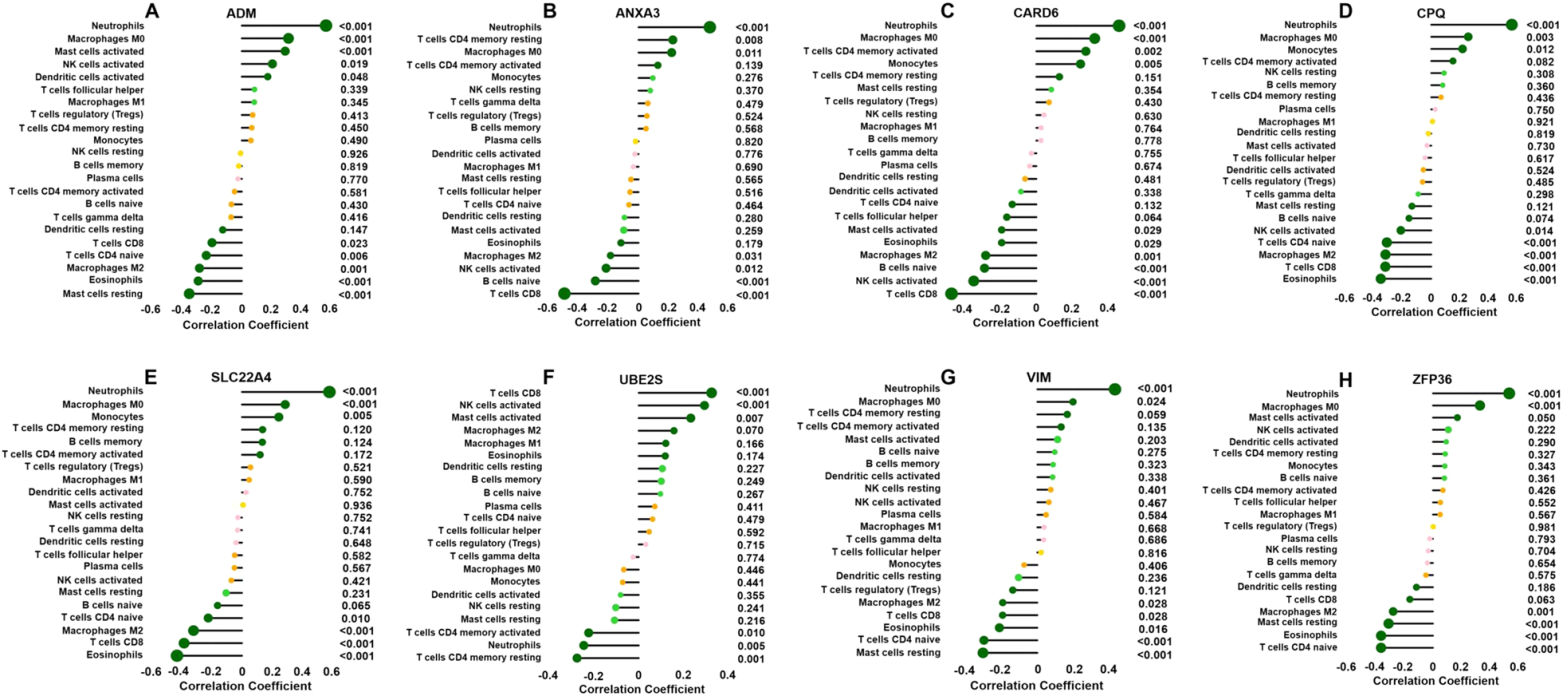
Correlation between *ADM* (**A**), *ANXA3* (**B**), *CARD6* (**C**), *CPQ* (**D**), *SLC22A4* (**E**), *UBE2S* (**F**), *VIM* (**G**), and *ZFP36* (**H**) and infiltrating immune cells. The size of the dots represents the strength of the correlation between the genes and immune cells; the larger the dots, the stronger the correlation. The colour of the dots represents the p value; the greener the colour, the lower the p value. *p* < 0.05 was considered statistically significant

As shown in Fig. 7, the *ADM* gene was positively correlated with M0 macrophages, neutrophils and activated mast cells and negatively correlated with resting mast cells, eosinophils and CD8 T cells; the *ANXA3* gene was positively correlated with M0 macrophages and neutrophils and negatively associated with naive B cells, CD8 T cells and activated NK cells; the *SLC22A4* gene was positively correlated with neutrophils, monocytes and M0 macrophages and negatively associated with eosinophils and CD8 T cells; the *VIM* gene was positively associated with neutrophils and M0 macrophages and negatively associated with resting mast cells, naive CD4 T cells, eosinophils and CD8 T cells; the *CARD6* gene was positively associated with neutrophils, monocytes and M0 macrophages and negatively associated with naive B cells, activated NK cells, eosinophils and CD8 T cells; the *CPQ* gene was positively associated with neutrophils, monocytes and M0 macrophages and negatively associated with naive CD4 T cells, eosinophils and CD8 T cells; and the *ZFP36* gene was positively associated with neutrophils and M0 macrophages and negatively associated with eosinophils, resting mast cells and naive CD4 T cells (*p* < 0.05–0.01, respectively).

### Validation of the key genes in the testing set and ischaemic stroke patients

**Fig. 8:**
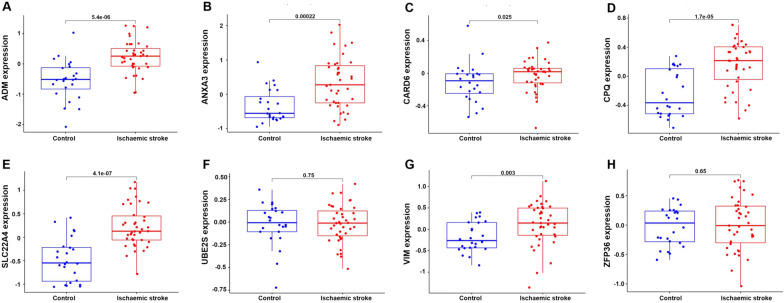
External validation of the key genes. The expression levels of *ADM* (**A**), *ANXA3* (**B**), *CARD6* (**C**), *CPQ* (**D**), *SLC22A4* (**E**), *UBE2S* (**F**), *VIM* (**G**) and *ZFP36* (**H**) in the GSE16561 testing set

**Fig. 9:**
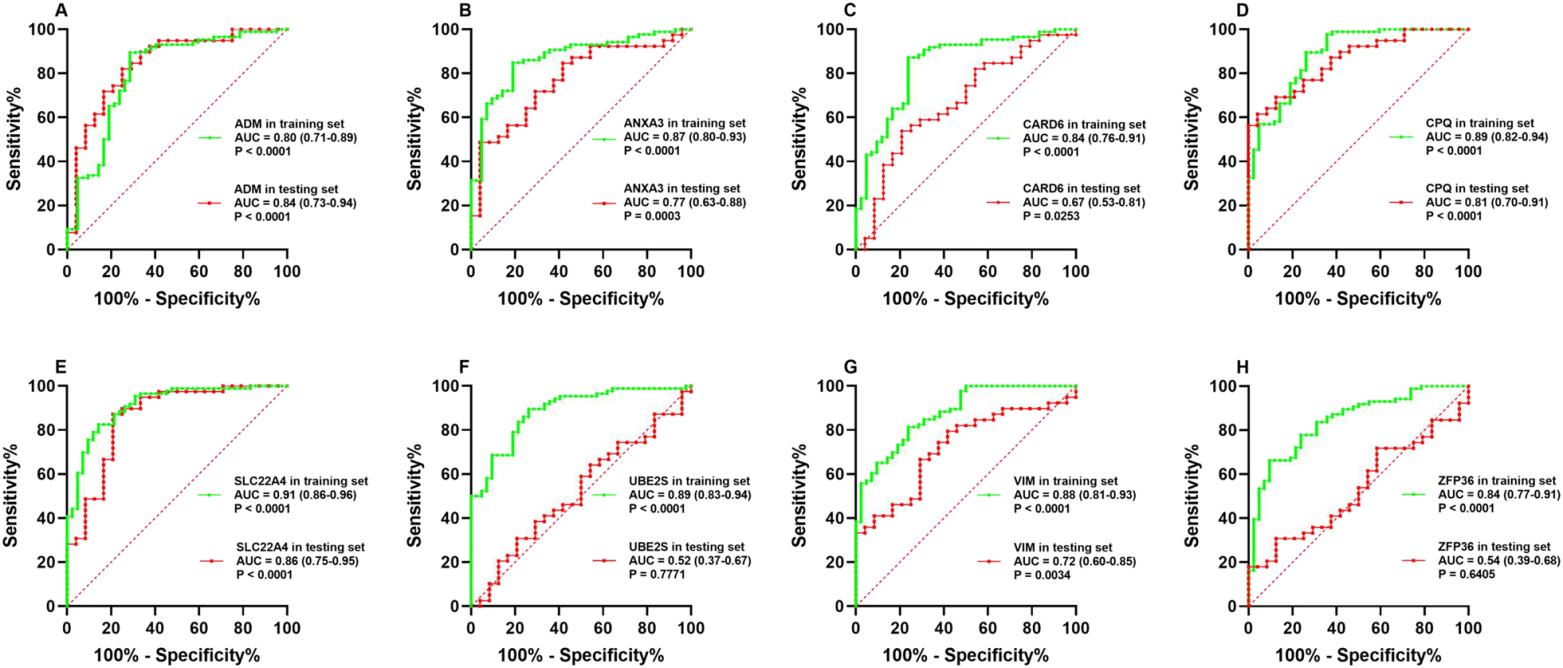
ROC curve analysis. ROC curve analysis of *ADM* (**A**), *ANXA3* (**B**), *CARD6* (**C**), *CPQ* (**D**), *SLC22A4* (**E**), *UBE2S* (**F**), *VIM* (**G**) and *ZFP36* (**H**) in the training set GSE22255 combined with GSE58294 (green line) and the testing set GSE16561 (red line)

As shown in Fig. 8, the expression levels of the *ADM*, *ANXA3*, *CARD6*, *CPQ*, *SLC22A4* and *VIM* genes were significantly increased in the ischaemic stroke patients compared with those in the healthy subjects (*p* < 0.05–0.01). However, the expression levels of the *UBE2S* and *ZFP36* genes did not significantly differ between the ischaemic stroke patients and normal subjects in the testing set. When no cross-validation is performed, the AUC values of *ADM*, *ANXA3*, *CARD6*, *CPQ*, *SLC22A4*, *UBE2S*, *VIM* and *ZFP36* were 0.80 (95% confidence interval (CI) 0.71–0.89), 0.87 (95% CI 0.80–0.93), 0.84 (95% CI 0.76–0.91), 0.89 (95% CI 0.82–0.94), 0.91 (95% CI 0.86–0.96), 0.89 (95% CI 0.83–0.94), 0.88 (95% CI 0.81–0.93) and 0.84 (95% CI 0.77–0.91) in the training set and 0.84 (95% CI 0.73–0.94), 0.77 (95% CI 0.63–0.88), 0.67 (95% CI 0.53–0.81), 0.81 (95% CI 0.70–0.91), 0.86 (95% CI 0.75–0.95), 0.52 (95% CI 0.37–0.67), 0.72 (95% CI 0.60–0.85) and 0.54 (95% CI 0.39–0.68) in the testing set, respectively (Fig. 9). When conducting 400 times of fivefold cross-validation, the mean and standard deviation of AUC of *ADM*, *ANXA3*, *CARD6*, *CPQ*, *SLC22A4*, *UBE2S*, *VIM* and *ZFP36* was 0.80 ± 0.09, 0.87 ± 0.07, 0.84 ± 0.08, 0.89 ± 0.07, 0.91 ± 0.06, 0.89 ± 0.06, 0.88 ± 0.07 and 0.84 ± 0.08 in the training set and 0.85 ± 0.11, 0.77 ± 0.13, 0.68 ± 0.13, 0.81 ± 0.12, 0.86 ± 0.11, 0.62 ± 0.11, 0.72 ± 0.13 and 0.61 ± 0.11 in the testing set, respectively.

**Fig. 10:**
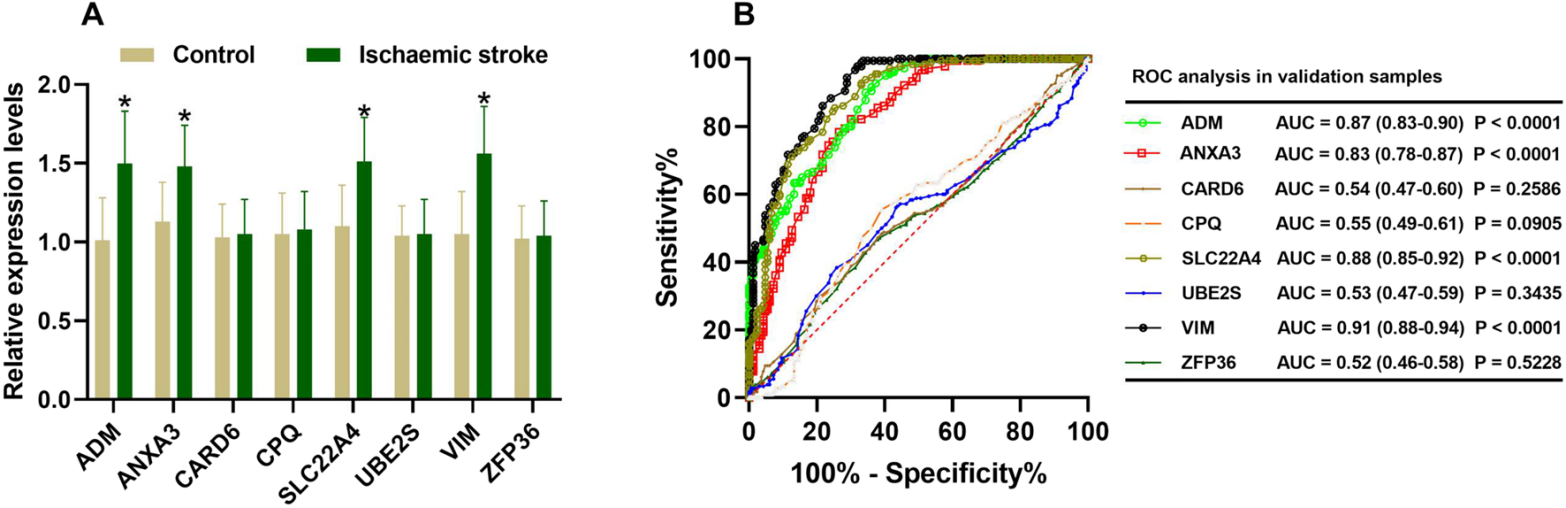
Validation of the key genes in ischaemic stroke samples. The relative expression levels of *ADM*, *ANXA3*, *CARD6*, *CPQ*, *SLC22A4*, *UBE2S*, *VIM* and *ZFP36* in ischaemic stroke patients (**A**). ROC curve analysis of *ADM*, *ANXA3*, *CARD6*, *CPQ*, *SLC22A4*, *UBE2S*, *VIM* and *ZFP36* based on the expression levels in ischaemic stroke patients (**B**)

Further verifying the expression levels of these key genes in ischaemic stroke patients, we noticed that the expression levels of *ADM*, *ANXA3*, *SLC22A4* and *VIM* were significantly increased in the ischaemic stroke patients compared with those in the normal subjects (*p* < 0.01). However, the expression levels of the *CARD6*, *CPQ*, *UBE2S* and *ZFP36* genes did not significantly differ between the ischaemic stroke patients and normal subjects (Fig. 10A). When no cross-validation is performed, the AUC values of *ADM*, *ANXA3*, *CARD6*, *CPQ*, *SLC22A4*, *UBE2S*, *VIM* and *ZFP36* were 0.87 (95% CI 0.83–0.90), 0.83 (95% CI 0.78–0.87), 0.54 (95% CI 0.47–0.60), 0.55 (95% CI 0.49–0.61), 0.88 (95% CI 0.85–0.92), 0.53 (95% CI 0.47–0.59), 0.91 (95% CI 0.88–0.94) and 0.52 (95% CI 0.46–0.58) in validation samples, respectively (Fig. 10B). When conducting 400 times of fivefold cross-validation, the mean and standard deviation of AUC of *ADM*, *ANXA3*, *CARD6*, *CPQ*, *SLC22A4*, *UBE2S*, *VIM* and *ZFP36* was 0.87 ± 0.04, 0.83 ± 0.04, 0.55 ± 0.05, 0.56 ± 0.06, 0.88 ± 0.04, 0.55 ± 0.05, 0.91 ± 0.03 and 0.54 ± 0.05 in validation samples, respectively.

### Demographic and biochemical characteristics

The patients with ischaemic stroke and those in the control group did not significantly differ in age, diastolic blood pressure, glucose, weight, body mass index (BMI), height, sex ratio, levels of total cholesterol (TC), apolipoprotein (Apo) B, proportion of smokers, hyperlipidaemia, Type 2 diabetes mellitus (T2DM) and alcohol consumption (Table 1). However, the ischaemic stroke patients had higher pulse pressure, systolic blood pressures, levels of serum low-density lipoprotein cholesterol (LDL-C), triglycerides (TGs) and proportions of hypertension than the healthy participants. In addition, the subjects in the control group had a markedly increased ApoA1/ApoB ratio, ApoA1, and serum high-density lipoprotein cholesterol (HDL-C) levels.

**Table 1:** Comparison of demographic, lifestyle characteristics and serum lipid levels of the participants

Characteristic	Control (n = 166)	Ischaemic stroke (n = 180)	Test‑statistic	*p*
Male/female ^c^	94/72	95/85	0.516	0.473
Age (years) ^a^	62.01 ± 9.40	63.25 ± 9.19	0.037	0.216
Height (cm) ^a^	160.45 ± 7.44	161.00 ± 7.47	0.832	0.495
Weight (kg) ^a^	56.94 ± 7.96	57.46 ± 8.88	3.245	0.569
BMI (kg/m^2^) ^a^	22.10 ± 2.61	22.16 ± 3.08	2.777	0.851
Smoking [n (%)] ^c^	66 (39.8)	75 (41.7)	0.130	0.718
Alcohol [n (%)] ^c^	43 (25.9)	50 (27.8)	0.154	0.694
SBP (mmHg) ^a^	128.70 ± 15.57	137.56 ± 19.09	28.524	< 0.001
DBP (mmHg) ^a^	74.63 ± 7.38	75.54 ± 10.38	13.634	0.351
PP (mmHg) ^a^	54.07 ± 15.31	62.01 ± 16.55	22.320	< 0.001
Glu (mmol/L) ^a^	6.02 ± 1.10	6.17 ± 1.30	0.662	0.247
TC (mmol/L) ^a^	4.28 ± 0.78	4.39 ± 0.79	0.026	0.198
TG (mmol/L) ^b^	0.93 (0.62)	1.16 (0.69)	-4.732	< 0.001
HDL-C (mmol/L) ^a^	1.86 ± 0.38	1.29 ± 0.36	1.316	< 0.001
LDL-C (mmol/L) ^a^	2.33 ± 0.72	2.71 ± 0.67	1.775	< 0.001
ApoA1 (g/L) ^a^	1.33 ± 0.22	1.03 ± 0.22	1.001	< 0.001
ApoB (g/L) ^a^	0.81 ± 0.18	0.81 ± 0.17	0.118	0.745
ApoA1/ApoB ^a^	1.73 ± 0.49	1.32 ± 0.39	8.560	< 0.001
Hyperlipidemia[n (%)]^c^	38 (22.9)	45 (25)	0.211	0.646
T2DM[n (%)]^c^	34 (20.5)	43 (22.9)	0.579	0.447
Hypertension[n (%)]^c^	40 (24.1)	82 (45.5)	14.873	< 0.001

*SBP* Systolic blood pressure; *DBP* Diastolic blood pressure; *PP* Pulse pressure; *Glu* Glucose; *HDL-C* high-density lipoprotein cholesterol; *LDL-C* low-density lipoprotein cholesterol; *Apo* Apolipoprotein; *TC* Total cholesterol; *TG* Triglyceride; T2DM Type 2 diabetes mellitus

^a^ Continuous data were presented as means ± SD and determined by two side *t*-test

^b^ Nonnormally distributed data were expressed using median and quartile ranges and were evaluated using the Wilcoxon-Mann–Whitney test

^c^ A chi-square analysis was used to evaluate the difference of the rate between the groups

## Discussion

In the current research, GSE22255 combined with GSE58294 as training datasets were downloaded from the GEO database and analysed using a WGCNA. Then, three modules (pink, brown and cyan) were identified to be significantly associated with ischaemic stroke. Eight hub genes (*ADM*, *ANXA3*, *CARD6*, *CPQ*, *SLC22A4*, *UBE2S*, *VIM* and *ZFP36*) were revealed to be significantly correlated with ischaemic stroke by a LASSO logistic regression and SVM-RFE machine learning methods. The CIBERSORT results revealed decreased infiltration of naive CD4 T cells, CD8 T cells, resting mast cells and eosinophils and increased infiltration of neutrophils, activated memory CD4 T cells and M0 macrophages in the ischaemic stroke patients. The external validation combined with the RT‒qPCR analysis revealed that the expression levels of *ADM*, *ANXA3*, *SLC22A4* and *VIM* were significantly increased in the patients with ischaemic stroke and that these key genes were positively correlated with M0 macrophages and neutrophils and negatively correlated with CD8 T cells. The ROC analyses based on the training set, validation set, and our clinical samples showed that the *ADM*, *ANXA3*, *SLC22A4* and *VIM* genes remained highly effective in distinguishing the ischaemic stroke patients from the normal subjects. These results suggest that the *ADM*, *ANXA3*, *SLC22A4* and *VIM* genes play a key role in the pathological process of ischaemic stroke.

Previous research has proven that the expression levels of adrenomedullin (*ADM*) are significantly increased in ischaemic cortical neurons induced by ischaemic injury in patients with ischaemic cerebrovascular disease [29]. Ischaemic cerebrovascular disease involves not only ischaemic brain cell injury but also arterial injury. Shinomiya et al. found that even in patients with fewer risk factors, the severity of atherosclerosis was significantly associated with elevated levels of mature ADM [30]. Ishikawa et al. proved that the expression levels of ADM were significantly increased in patients with unstable coronary artery disease compared with those in patients suffering from stable coronary artery disease, and ADM may participate in the instability of atherosclerotic plaque in the form of autocrine or paracrine [31]. Matthew et al. proved that ADM acts as an independent predictor of major adverse cardiovascular events (MACEs) in patients suffering from heart failure and acute myocardial infarction (AMI), and the quantification of the ADM levels may help improve the risk stratification of heart failure and myocardial infarction [32]. In addition, a compelling study showed that elevated ADM levels were significantly associated with the severity of neurological damage, higher mortality, and poorer outcomes in patients with ischaemic stroke [33].

Through a comprehensive search of the NCBI GENE database, we revealed that Annexin A3 (*ANXA3*, also known as *ANX3*; HGNC: 541, gene ID: 306, OMIM: 106,490) is located on chromosome 4q21.21 (exon count: 14), acts as a member of the annexin family, and plays a crucial role in regulating multiple biological processes, such as inflammatory responses, cell proliferation, apoptosis and tumorigenesis [34]. Junker et al. [35] and Kessler et al. [36] reported that the expression levels of ANXA3 were significantly upregulated in the infarcted area after cerebral ischaemia injury in rats. Hua et al. proved that silencing the ANXA3 gene can promote the repair and healing of ischaemic myocardium by activating the PI3K/Akt signalling pathway in rats with AMI [37]. Moreover, Min et al. found that miR-18b can protect cerebral ischaemia‒reperfusion injury by activating the PI3K/Akt signalling pathway by inhibiting the expression of ANXA3 [38].

Solute carrier family 22 member 4 (*SLC22A4,* also known as *OCTN1*; DFNB60, gene ID: 5583, HGNC: 10,968, OMIM: 604,190) is located on chromosome 5q31.1 (exon count: 11) and encodes an organic cation transporter across the plasma membrane of epithelial cells. Previous research showed that the SLC22A4 variant, as an inflammation-related gene polymorphism involved in the innate immune response, is significantly correlated with an increased susceptibility to inflammatory bowel disease (IBD), Crohn's disease (CD) and ulcerative colitis (UC) by changing the transcription and function of organic cation transporters [39–41]. Meanwhile, the genetic polymorphisms SLC22A4 rs2073838 and rs3792876 were reported to be significantly associated with rheumatoid arthritis (RA) in the Japanese population [42] and Chinese population [43]. Tokuhiro et al. also suggested that SLC22A4 was significantly overexpressed in the inflammatory joints of mice with collagen-induced arthritis, and runt-related transcription factor 1 (RUNX1) can affect the susceptibility to RA by regulating the expression of SLC22A4 [44]. McCann et al. observed that inappropriate triggering of the inflammatory response can be effectively reduced by reducing the abnormal transport function of the SLC22A4 503F variant [45]. In addition, Yamase et al. proved that the genetic polymorphisms of SLC22A4 rs273909 were significantly associated with ischaemic stroke in the Japanese population [46].

Vimentin (*VIM*, gene ID: 100,507,347, HGNC: 44,879, OMIM: 193,060) acts as a cytoskeletal intermediate silk protein, plays a crucial role in neuritogenesis and cholesterol transport, and functions as an organizer of several key proteins involved in subsequent biological processes, such as signal transduction, adhesion, migration, apoptosis, and differentiation [47]. Kim et al. found that oxidized low density lipoprotein (ox-LDL) can induce the synthesis and secretion of VIM in macrophages, while extracellular VIM can induce macrophages to release inflammatory cytokines, such as tumour necrosis factor-α (TNF-α) and interleukin 6 (IL-6), which subsequently lead to atherosclerotic inflammation [48]. He et al. proved that silencing the expression of miR-144 can significantly promote the expression of VIM and the formation of atherosclerotic plaques [49]. Yao et al. suggested that inhibiting the expression and rearrangement of VIM can effectively reduce the migration of vascular smooth muscle cells induced by TNF-α, thereby alleviating the progression of atherosclerotic lesions [50]. Gong et al. found that the serum VIM levels were higher in patients with coronary artery disease (CAD), and the VIM levels were positively correlated with the severity of CAD. In addition, these authors found that VIM can accelerate the occurrence and development of atherosclerotic lesions by inducing macrophages to secrete proinflammatory cytokines and adhesion molecules [51]. Furthermore, Xiao et al. found that VIM can increase the instability of plaques, and an elevated level of VIM can significantly increase the risk of ischaemic stroke in patients with carotid plaques [52].

Adverse innate immune responses are associated with several disease processes. Fernandez et al. provided the first systematic description of the morphology of immune cells during atherosclerosis, provided insight into which immune cells reside in plaques and described their different activation states, which opened the door to the study of atherosclerosis caused by the immune response [53]. Monocyte subsets play a crucial role in the atherogenesis and inflammatory cascade of cardiovascular disease. Upregulated counts and monocyte activity are significantly related to clinical indices of chronic kidney disease (CKD) and atherosclerosis [54]. T lymphocytes, which act as the most important type of immune cells, can be divided into CD4 and CD8 cell subsets according to their surface markers and functions. CD8 T cells play a dual role in atherosclerosis. Previous studies have suggested that CD8 T cells can secrete various inflammatory cytokines, which can aggravate the inflammatory response and increase the instability of atherosclerotic plaques [55]. However, cytotoxic activity targeting antigen presenting cells and regulatory CD8 T cells could effectively inhibit the progression of atherosclerosis by alleviating the immune reaction [55]. Other immune cell types, including neutrophils [56] and master cells [57], also play a key role in the occurrence and development of cardiovascular disease. Furthermore, Li et al. found that the proportion of M1 macrophages, gamma delta (γδ) T cells and neutrophils was significantly higher and that the proportion of eosinophils and resting dendritic cells was significantly lower in ischaemic stroke patients compared to those in healthy subjects. However, the immune infiltration pattern of ischaemic stroke has not been fully elucidated. Clarifying immune infiltration in ischaemic stroke and identifying the key genes related to immune cells could provide a new perspective for the prevention and treatment of ischaemic stroke.

To further evaluate the proportion and type of immune cell infiltration in ischaemic stroke, the CIBERSORT package in R was utilized to carry out a comprehensive assessment of 22 types of immune cell infiltration in ischaemic stroke patients. We noticed that there was a decreased infiltration of naive CD4 T cells, CD8 T cells, resting mast cells and eosinophils and an increased infiltration of neutrophils, M0 macrophages and activated memory CD4 T cells in ischaemic stroke patients. As previously mentioned, the inflammatory characteristics of circulating neutrophils were increased in the acute stage of ischaemic stroke, and activated neutrophils may promote the progression of ischaemic stroke by promoting systemic inflammation and destroying the blood‒brain barrier [58]. Compared with the normal samples, the proportion of neutrophils in the ischaemic stroke samples was generally higher; neutrophils are involved in ischaemic injury after stroke and may be a promising target for ischaemic stroke therapies [59]. In addition, CD8 T cells play a dual role in atherosclerosis, and our study showed that the proportion of neutrophils was higher while the proportion of CD8 T cells was lower in the ischaemic stroke patients compared with those in the control subjects. This finding implies that neutrophils can accelerate but CD8 T cells can inhibit the occurrence and progression of ischaemic stroke. However, whether the number of CD8 T cells and neutrophils in peripheral blood samples could reflect their infiltration pattern in the vascular wall remains unclear. In addition, the current research revealed the interaction of 22 types of infiltrated immune cells in ischaemic stroke. Neutrophils were negatively associated with CD8 T cells and eosinophils and positively associated with M0 macrophages. Moreover, these key genes including *ADM*, *ANXA3*, *SLC22A4* and *VIM* were positively correlated with M0 macrophages and neutrophils and negatively correlated with CD8 T cells. However, a large number of studies have shown that immune checkpoint inhibitors targeting programmed cell death 1 (PD1), programmed cell death ligand 1 (PDL1) and cytotoxic T-lymphocyte associated protein 4 (CTLA4) can effectively improve the prognosis of many cancer patients, but it may lead to some vascular and cardiac toxicity such as atherosclerosis, ischaemic stroke or myocardial infarction and other adverse reactions [7, 60, 61]. Therefore, further studies are needed to explore whether therapies targeting these genes such as *ADM*, *ANXA3*, *SLC22A4* and *VIM* will bring some similar risks to patients with ischaemic stroke.

On the other hand, the gene enrichment analysis indicated that these key genes were mainly involved in inflammatory or immune-related signalling pathways, such as the NF-kappa B (NF-κB) signalling pathway, TNF signalling pathway, Toll-like receptor signalling pathway, NOD-like receptor signalling pathway and IL-17 signalling pathway. Previous studies have shown that the transcription factor NF-κB is a main regulator of genes involved in the inflammatory response [60, 62], and NF-κB has been shown to play an important role in ADM-induced inflammation [62]. The overexpression of NF-κB can participate in the rheumatoid arthritis-related inflammatory response by activating the SLC22A4 promoter [63]. The inhibition of NF-κB can reduce the expression of VIM and affect the epithelial mesenchymal transformation and nerve infiltration in pancreatic cancer [64]. In addition, Liu et al. suggested that the NF-κB signalling pathway plays a key role in the biological processes of cell proliferation, migration and apoptosis mediated by ANXA3 [34]. These findings are consistent with our bioinformatics analysis and suggest that the NF-κB signalling pathway plays an important role in the biological processes mediated by these key genes, including *ADM*, *ANXA3*, *SLC22A4* and *VIM.* However, the regulatory relationship among these key genes, the NF-κB signalling pathway and the mechanism of action in ischaemic stroke still need further experimental verification.

This research had several limitations. First, the RT‒qPCR analysis found that there was no significant difference in the expression levels of *CARD6*, *CPQ*, *UBE2S* and *ZFP36* between our ischaemic stroke patients and normal subjects. The validation samples included in the current research were recruited from only a single centre with small sample sizes. Whether the expression levels of the above genes differ among individuals in different regions or races is unclear. Therefore, the results of this study need to be further tested in multicentre studies with larger samples. Second, whether the number of CD8 T cells and neutrophils in peripheral blood samples could reflect their infiltration in the vascular wall remains unclear. Third, more in vivo and in vitro studies are needed to clarify the underlying mechanism of these correlations among *ADM*, *ANXA3*, *SLC22A4* and *VIM* and infiltrated immune cells in ischaemic stroke.

## Conclusions

In summary, we determined that *ADM*, *ANXA3*, *SLC22A4* and *VIM* are diagnostic markers of ischaemic stroke. We noticed that neutrophils, activated memory CD4 T cells and M0 macrophages may be related to the initiation and progression of ischaemic stroke; however, naive CD4 T cells, resting mast cells, CD8 T cells and eosinophils play a protective role in ischaemic stroke. This paper also indicates that *ADM*, *ANXA3*, *SLC22A4* and *VIM* are positively correlated with neutrophils and M0 macrophages and negatively correlated with CD8 T cells*.* The mechanism underlying the relationship between *ADM*, *ANXA3*, *SLC22A4* and *VIM* and immune cells may be of great significance for the pathogenesis and progression of ischaemic stroke, and related research of these genes could provide new therapeutic insight for ischaemic stroke.

## Supplementary Information

**Additional file 1. **Figure S1. Clustering dendrogram of samples. Figure S2. Infiltration pattern of immune cell subtypes in validation set.

**Additional file 2. **Table S1. The expression profile of the top 25% of genes with high expression variance in the training set (GSE22255 combined with GSE58294).

**Additional file 3. **Table S2. The expression profile of the 17468 genes in the testing set (GSE16561).

**Additional file 4. **Table S3. The clinical features of the 128 samples in the training set (GSE22255 combined with GSE58294).

**Additional file 5. **Table S4. The GS values as well as corresponding p values of 519 genes in the pink, brown and cyan modules.

**Additional file 6. **Table S5. Detailed results of the KEGG and GO enrichment analysis.

**Additional file 7. **Table S6. The key genes identified by the LASSO regression and/or SVM-RFE algorithm.

**Additional file 8.**Table S7. The immune cell infiltration pattern of 128 ischaemic stroke samples in the training set (GSE22255 combined with GSE58294).

## Data Availability

The raw data supporting the conclusions of this article will be made available by the authors, without undue reservation.

## Abbreviations

WGCNA: Weighted gene co-expression network analysis
GS: Gene significance
SVM-RFE: Support vector machine-recursive feature elimination
AMI: Acute myocardial infarction
LASSO: Least absolute shrinkage and selection operator
CT: Computed tomography
MRI: Magnetic resonance imaging
T2DM: Type 2 diabetes mellitus
GO: Gene Ontology
HDL-C: High-density lipoprotein cholesterol
IS: Ischemic stroke
KEGG: Kyoto Encyclopedia of Genes and genomes
LDL-C: Low-density lipoprotein cholesterol
MACEs: Major adverse cardiovascular events
Apo: Apolipoprotein
GEO: Gene Expression Omnibus
BMI: Body mass index
TG: Triglyceride
RT-qPCR: Quantitative real time polymerase chain reaction
TC: Total cholesterol
TOM: Topological overlap matrix
Mes: Module eigengenes
MM: Module membership
CD: Crohn's disease
UC: Ulcerative colitis
AUCs: Areas under the curves
IBD: Inflammatory bowel disease
RA: Rheumatoid arthritis
RUNX1: Runt-related transcription factor 1
ox-LDL: Oxidized low density lipoprotein
CAD: Coronary artery disease
MM: Module membership
IL-6: Interleukin 6
CKD: Chronic kidney disease
γδ: Gamma delta
ADM: Adrenomedullin
ANXA3: Annexin A3
CARD6: Caspase recruitment domain family member 6
CPQ: Carboxypeptidase Q
SLC22A4: Solute carrier family 22 member 4
UBE2S: Ubiquitin conjugating enzyme E2 S
VIM: Vimentin
ZFP36: ZFP36 ring finger protein
PD1: Programmed cell death 1
PDL1: Programmed cell death ligand 1
CTLA4: Cytotoxic T-lymphocyte associated protein 4
