# Supplementary material for: Identification of immune-related key genes in the peripheral blood of ischaemic stroke patients using a weighted gene coexpression network analysis and machine learning
Source: J Transl Med. 2022 Aug 12;20:361. doi: 10.1186/s12967-022-03562-w (PMC9373395; doi:10.1186/s12967-022-03562-w)
Supplement: Supplementary file 1 — Additional file 1. Figure S1. Clustering dendrogram of samples. Figure S2. Infiltration pattern of immune cell subtypes in validation set. [file 12967_2022_3562_MOESM1_ESM.docx]

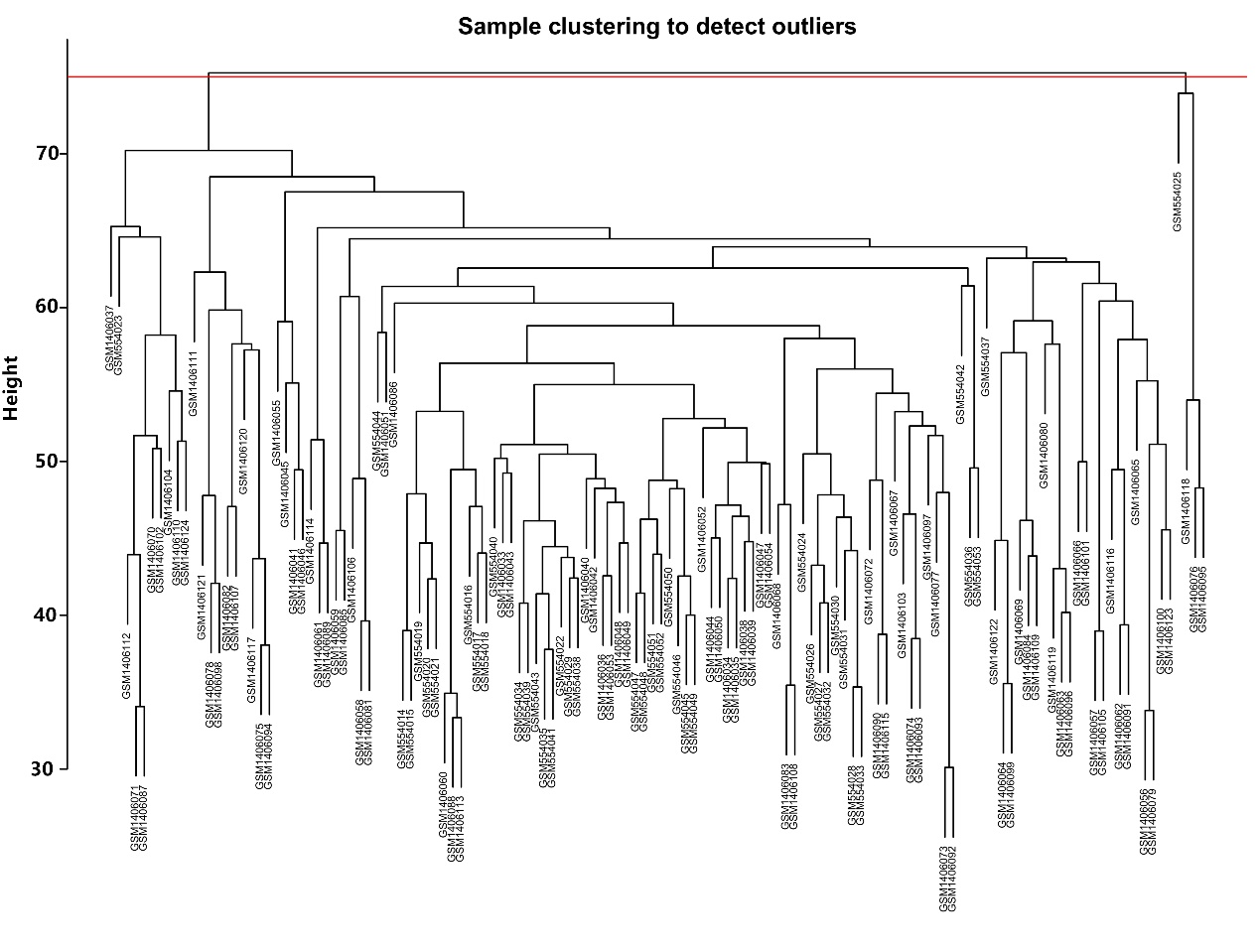


**Additional** **Figure S1: Clustering dendrogram of samples.**

Cut height = 75 was used to divide 132 samples into two different cluster types. Cluster 1contains the following samples (GSM554014, GSM554015, GSM554016, GSM554017, GSM554018, GSM554019, GSM554020, GSM554021, GSM554022, GSM554023, GSM554024, GSM554026, GSM554027, GSM554028, GSM554029, GSM554030, GSM554031, GSM554032, GSM554033, GSM1406033, GSM1406034, GSM1406035, GSM1406036, GSM1406037, GSM1406038, GSM1406039, GSM1406040, GSM1406041, GSM1406042, GSM1406043, GSM1406044, GSM1406045, GSM1406046, GSM1406047, GSM1406048, GSM1406049, GSM1406050, GSM1406051, GSM1406052, GSM1406053, GSM1406054, GSM1406055, GSM554034, GSM554035, GSM554036, GSM554037, GSM554038, GSM554039, GSM554040, GSM554041, GSM554042, GSM554043, GSM554044, GSM554045, GSM554046, GSM554047, GSM554048, GSM554049, GSM554050, GSM554051, GSM554052, GSM554053, GSM1406056, GSM1406057, GSM1406058, GSM1406059, GSM1406060, GSM1406061, GSM1406062, GSM1406063, GSM1406064, GSM1406065, GSM1406066, GSM1406067, GSM1406068, GSM1406069, GSM1406070, GSM1406071, GSM1406072, GSM1406073, GSM1406074, GSM1406075, GSM1406077, GSM1406078, GSM1406079, GSM1406080, GSM1406081, GSM1406082, GSM1406083, GSM1406084, GSM1406085, GSM1406086, GSM1406087, GSM1406088, GSM1406089, GSM1406090, GSM1406091, GSM1406092, GSM1406093, GSM1406094, GSM1406096, GSM1406097, GSM1406098, GSM1406099, GSM1406100, GSM1406101, GSM1406102, GSM1406103, GSM1406104, GSM1406105, GSM1406106, GSM1406107, GSM1406108, GSM1406109, GSM1406110, GSM1406111, GSM1406112, GSM1406113, GSM1406114, GSM1406115, GSM1406116, GSM1406117, GSM1406119, GSM1406120, GSM1406121, GSM1406122, GSM1406123, GSM1406124), cluster 2 contains the following samples (GSM554025, GSM1406118, GSM1406076 and GSM1406095)


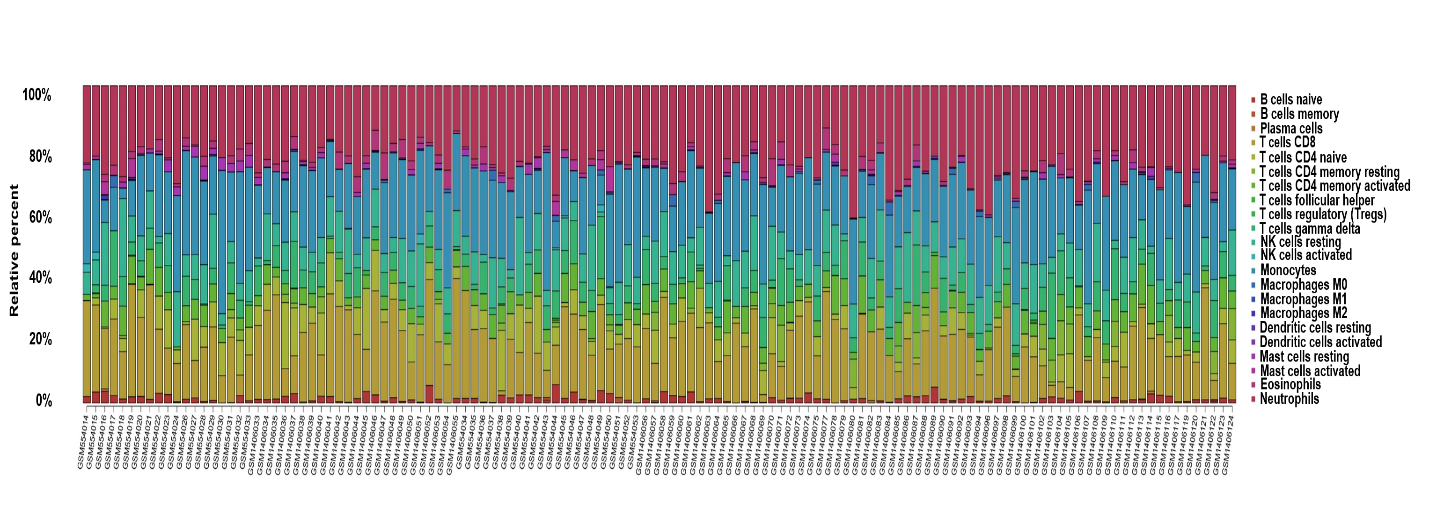


**Additional Figure S2: Infiltration pattern of immune cell subtypes in validation set.**

The bar plot visualizing the relative percent of 22 immune cell in each sample.
